# Risk factors for arteriovenous fistula dysfunction in hemodialysis patients: a retrospective study

**DOI:** 10.1038/s41598-023-48691-4

**Published:** 2023-12-03

**Authors:** Fan Zhang, Jiali Li, Jie Yu, Yang Jiang, Hailang Xiao, Yiya Yang, Yumei Liang, Kanghan Liu, Xun Luo

**Affiliations:** 1 Department of Nephrology and Laboratory of Kidney Disease, Hunan Provincial People’s Hospital, The First Affiliated Hospital of Hunan Normal University, Changsha, China; 2 Department of Nephrology, Changsha central hospital, Changsha, China

**Keywords:** Renal replacement therapy, Haemodialysis

## Abstract

Arteriovenous fistula (AVF) is the first choice of vascular access in hemodialysis (HD) patients. However, the correlations between patient factors and the arteriovenous fistula patency remain unclear. Therefore, our study investigates the risk factors associated with AVF dysfunction in HD patients. A total of 233 end-stage renal disease (ESDR) patients who met the study inclusion criteria in the Nephrology Department of Hunan Provincial People’s Hospital between December 2020 and June 2022 were included in this study. The baseline demographic, clinical and laboratory parameters were collected at the time of AVF creation and analyzed. Of the 233 ESRD patients, 146 (62.7%) were male and the mean age was 56.11 ± 12.14 (21–82) years. The patients were followed for a median time of 14 months. Kaplan–Meier analysis showed a 6-, 12- and 24-month post-placement survival of 87.1%, 82.8% and 80.7%, respectively. Univariate Cox regression analysis revealed weight (HR, 1.03; *P* = 0.03) as a predictor for the loss of vascular access functionality. In addition, multivariate Cox regression analysis further demonstrated that sex (HR, 3.41; *P* = 0.03), weight (HR 1.08; *P* < 0.01) and phosphorus level (HR: 3.03; *P* = 0.01) are independent risk factors for AVF dysfunction. AVF dysfunction is highly associated with several risk factors including weight, phosphorus level, and sex. Positive intervention strategies targeting these potential factors, such as weight loss or oral phosphate binders could improve the long-term success of AVF.

## Introduction

The increasing prevalence of end-stage renal disease (ESRD) has led to a steep rise in the number of patients requiring hemodialysis (HD). Vascular access (VA) is required for the well-being and survival of HD patients and has been referred to as both the “lifeline” and “Achilles’ heel” for HD patients^1^. There are three types of VA, namely arteriovenous fistula (AVF), arteriovenous graft (AVG), and central venous catheters (CVC). AVF is the optimal VA for HD due to its longevity, lower rates of infection and thrombosis, and greater safety compared with AVG or CVC^2,3^.

Although the merits of AVF make it a preferred form of renal replacement therapy, it has been revealed that 20–50% of AVFs would fail to mature adequately to vascular access for hemodialysis^4^. AVF failure consists of three types, including early thrombosis, failure to mature, and late failure^5^. The characteristic pathology of AVF failure included neointimal hyperplasia, failure to develop outward remodeling or wall thicking^5^. Accordingly, a unique set of biochemical abnormalities may predispose the vascular wall to inward remodeling and stenosis after AVF creation, including obesity, chronic inflammation, CKD-MBD, hyperphosphatemia, endothelial failure, and lipidemia. In addition, many other variables are also associated with AVF failure, such as patient’s sex, age and comorbidities. However, the exact role of these factors in AVF failure is not completely well defined yet.

In this retrospective study, we aimed to assess the risk factors associated with AVF dysfunction in HD patients, providing new insights for the prevention of AVF failure.

## Material and methods

### Study design

**Figure 1:**
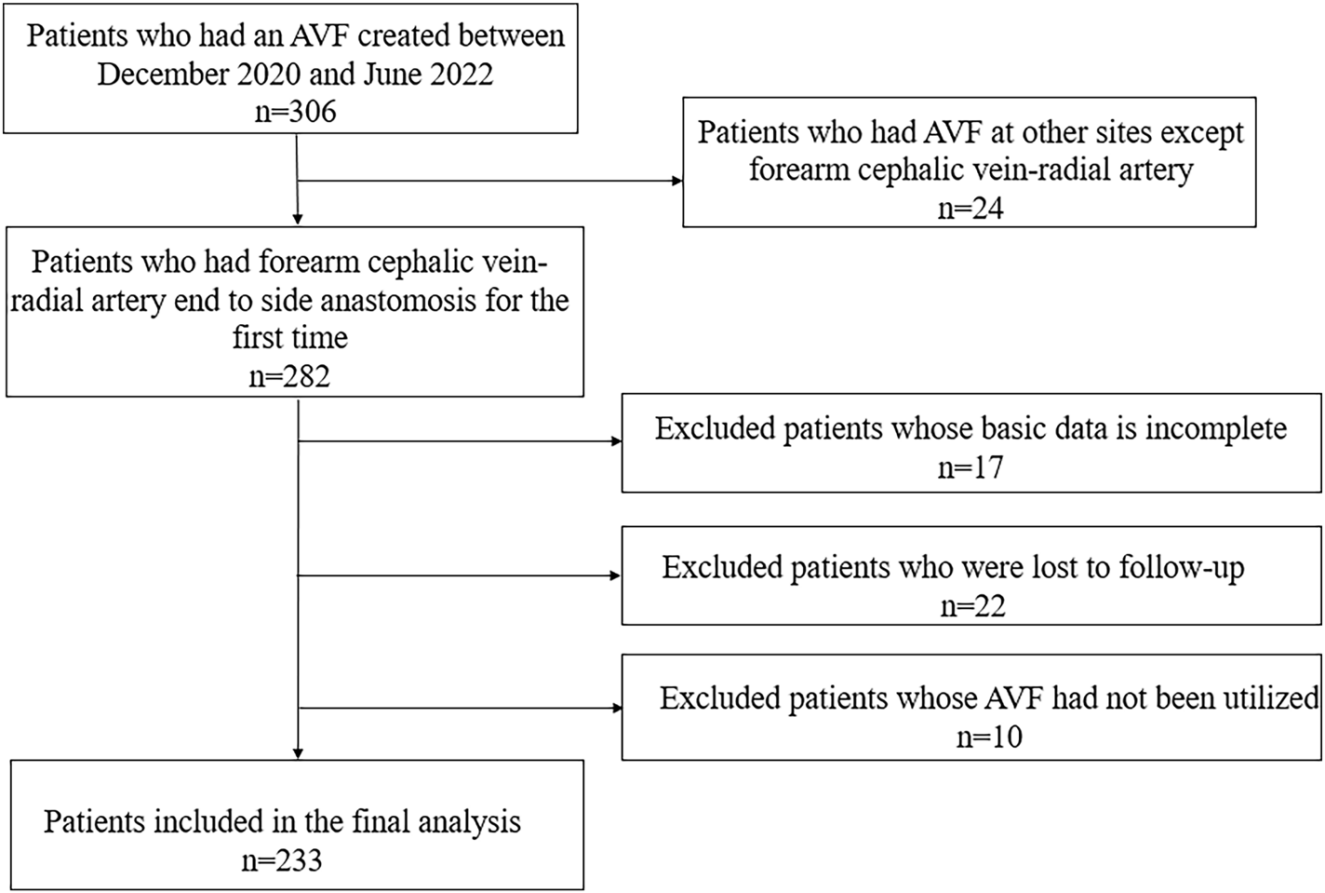
Patient screening flow chart.

The AVF patency rate of ESRD patients who had an AVF created between December 2020 and June 2022 at the Nephrology Department of Hunan Provincial People’s Hospital was retrospectively analyzed. A total of 306 ESRD patients were enrolled and assessed for eligibility. Inclusion criteria were listed as follows: (1) Age ≥ 18 years at the time of fistula establishment; (2) ESRD patients who underwent forearm cephalic vein-radial artery end to side anastomosis for the first time; (3) The success of first AVF maturation (blood flow ≥ 200 mL/min) and received initial routine HD (2–3 times per week). Exclusion criteria were listed as follows: (1) Patients who were unable to participate telephone or in-person follow-up for any reason; (2) Patients whose basic data could not be collected; (3) Patients whose AVF had not been utilized.A final total of 233 ESRD patients who met the eligibility criteria and agreed to participate were included in the study (Fig. 1). The demographics and clinical data of the patients were collected at the time of AVF creation. The patients were followed by telephone or in-person, and relevant data were collected from the patients’ records. Patients were divided into patency (n = 191) and dysfunction (n = 42) groups based on whether their AVF was patent or not. AVF dysfunction was defined as lower blood flow during dialysis (≤ 200 mL/min).The study involving human participants adhered to ethical standards set by the institution and the national research committee. It was in accordance with the principles outlined in the 1964 Helsinki Declaration and its later amendments or similar ethical standards. All patients provided written informed consent and the Ethics Committee of Hunan Provincial People’s Hospital approved the study.

### Demographic, clinical, and laboratory variables

The demographic, clinical, and laboratory characteristics of the patients were collected from their medical records at the time of AVF creation. These data included personal data (age and sex), smoking status, anthropometric data (height, weight, and body mass index), primary disease of ESRD, preoperative blood pressure, and other comorbidities (hypertension and diabetes). The initial laboratory workup included complete blood count, albumin, uric acid, creatinine, cholesterol, triglycerides, calcium, phosphorus, intact parathyroid hormone, and ferritin. All blood specimens were collected on the day before arteriovenous fistula plasty.

### Statistical analysis

All data were analyzed using SPSS 19.0. Continuous data are expressed as mean ± standard deviation and compared using the independent *t* test (normal distribution) or Mann–Whitney *U* test (non-normal distribution). Categorical variables are reported in frequency and percentage, and compared using the chi-square test. AVF patency was calculated by the Kaplan–Meier method, and risk factors for AVF dysfunction were identified by univariate and multivariate Cox regression analyses. A *P* < 0.05 was considered statistically significant.

## Results

**Figure 2:**
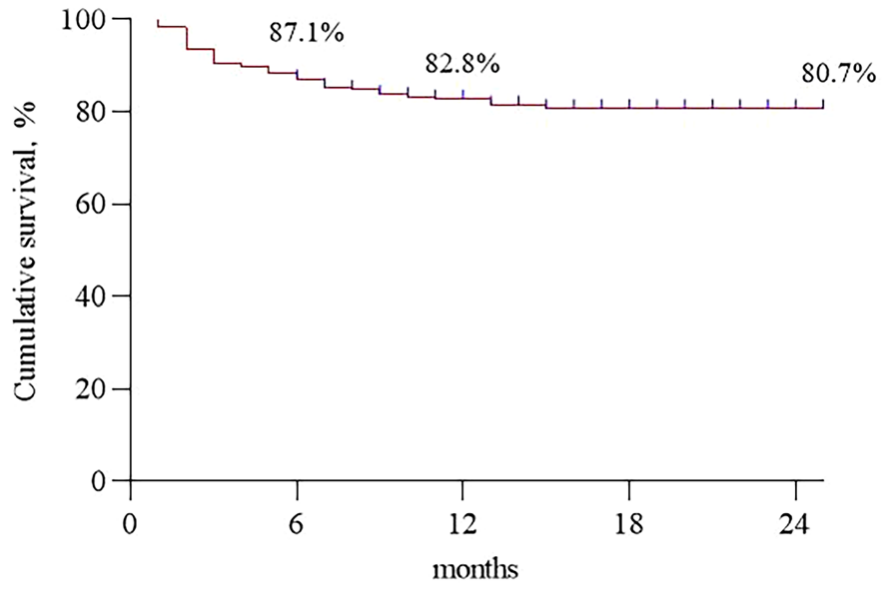
Cumulative survival of arteriovenous fistula.

Of the 233 ESRD patients, 146 (62.7%) were male and the mean age was 56.11 ± 12.14 years. The patients were followed for a median time of 14 months. Kaplan–Meier analysis showed the cumulative AVF survival was 87.1% at 6 months, 82.8% at 12 months, and 80.7% at 24 months (Fig. 2). No death was recorded in the follow-up period.

The baseline demographic and clinical characteristics of the patients in the patency group (n = 191) and AVF dysfunction group (n = 42) are summarized in Table 1. Among patients in the AVF dysfunction group, 24 (57.1%) were male, and the mean age was 57.43 ± 12.41 (25–82) years. In the normal AVF group, 122 (63.9%) were male and the mean age was 55.82 ± 12.10 (21–76 years). A total of 20 parameters were collected and compared between the two groups, including weight, diastolic pressure, albumin, and uric acid (Table 2). Compared with the patency group, patients in the AVF dysfunction group had greater weight (68.97 ± 12.23 vs. 63.79 ± 11.61; *P* = 0.02), lower diastolic pressure (86.10 ± 11.00 vs. 79.71 ± 14.66; *P* < 0.01) (Table 1) and lower albumin (33.69 g/L vs. 30.29 g/L; *P* = 0.01) and uric acid (493.0 mmol/L vs. 437.5 mmol/L; P = 0.03) levels. Prothrombin activity (PTA) was significantly higher in AVF patency group than that in AVF dysfunction group (*P* = 0.04). No significant differences in remaining parameters was detected between the two groups.

**Table 1:** Baseline characteristics of AVF patency and dysfunction group.

Characteristics	Overall (n = 233)	Patency (n = 191)	Dysfunction (n = 42)	t/${\chi }^{2}$	*P*
Sex					
Male	146	122 (63.9)	24 (57.1)	0.67	0.41
Female	87	69 (36.1)	18 (42.9)		
Age (year)	56.11 ± 12.14	55.82 ± 12.10	57.43 ± 12.41	− 0.78	0.44
Height (m)	1.64 ± 0.07	1.64 ± 0.07	1.63 ± 0.06	0.17	0.87
Weight (kg)	64.61 ± 11.83	63.79 ± 11.61	68.97 ± 12.23	− 2.30	**0.02**
SBP (mmHg)	84.96 ± 11.95	86.10 ± 11.00	79.71 ± 14.66	3.16	** < 0.01**
DBP (mmHg)	157.32 ± 25.13	158.11 ± 24.20	153.66 ± 29.14	1.03	0.30
Smoking	29	26 (13.6)	3 (7.1)	1.32	0.25
Comorbidities					
Hypertension	198	164 (85.9)	34 (81.0)	0.65	0.42
Diabetes mellitus	84	71 (37.2)	13 (31.0)	0.58	0.45
Coronary artery disease	104	84 (44.0)	20 (47.6)	0.19	0.67
Peripheral vascular disease	25	19 (9.9)	6 (14.3)	0.68	0.41
Heart failure	63	51 (26.7)	12 (28.6)	0.06	0.81
Cause of CKD				1.59	0.66
Glomerulonephritis	109	90 (47.1)	19 (45.2)		
Others	114	93 (48.7)	21 (50.0)		
Unkown	8	7 (3.7)	1 (2.4)		
Medication					
Antiplatelet therapy	43	31 (16.2)	12 (28.6)	3.48	0.06
Beraprost	28	22 (11.5)	6 (14.3)	0.25	0.62
Statins	61	49 (25.7)	12 (28.6)	0.15	0.70

*DBP* diastolic blood pressure, *SBP* systolic blood pressure, *CKD* chronic kidney disease.

Categorical variables are presented as number (%). Continuous variables with Gaussian distribution are presented as mean ± standard deviations, s.

P for comparison between patients with *AVF* patency and *AVF* dysfunction.

**Table 2:** Biologic characteristics of AVF patency and dysfunction group.

Characteristic	Overall (n = 233)	Patency (n = 191)	Dysfunction (n = 42)	Z/t	P
TC (mmol/L)	4.09 (3.33, 5.01)	4.12 (3.39, 4.93)	3.84 (3.19, 5.48)	− 0.05	0.96
TG (mmol/L)	1.44 (0.97, 2.07)	1.44 (0.97, 2.10)	1.43 (0.97, 1.92)	− 0.24	0.81
HDL (mmol/L)	0.99 (0.82, 1.27)	0.99 (0.82, 1.25)	1.00 (0.76, 1.29)	− 0.10	0.92
LDL (mmol/L)	1.98 (1.44, 2.69)	1.99 (1.46, 2.69)	1.81 (1.00, 3.08)	− 0.86	0.39
PLT (× 10^9^/L)	195.59 ± 82.21	193.57 ± 79.93	204.74 ± 92.32	− 0.80	0.43
MPV (fl)	10.24 ± 1.46	10.19 ± 1.42	10.46 ± 1.63	− 1.11	0.27
PDW (fl)	15.60 (11.20, 41.40)	15.60 (11.20, 42.50)	15.45 (10.85, 18.35)	− 0.08	0.93
WBC (× 10^9^/L)	6.60 (4.95, 8.43)	6.77 (4.85, 8.45)	6.37 (5.33, 7.35)	− 0.33	0.74
PTH (pg/ml)	263.7 (147.0, 446.6)	277.2 (153.1, 450.0)	224.1 (120.4, 374.6)	− 1.31	0.19
NEU (× 10^9^/L)	4.82 (3.39, 6.32)	4.89 (3.38, 6.32)	4.62 (3.42, 6.15)	− 0.30	0.77
MONO (× 10^9^/L)	0.43 (0.31, 0.56)	0.43 (0.31, 0.56)	0.46 (0.34, 0.57)	− 0.72	0.47
LYMPH (× 10^9^/L)	0.90 (0.70, 1.26)	0.90 (0.71, 1.27)	0.85 (0.70, 1.25)	− 0.40	0.67
NLR	4.70 (3.36, 7.76)	4.75 (3.19, 7.90)	4.56 (3.69, 7.01)	− 0.01	1.00
PLR	195.6 (134.7, 278.1)	184.5 (134.0, 271.4)	217.2 (148.3, 304.5)	− 1.08	0.28
β2MG (mg/L)	21.35 (16.46, 26.73)	21.24 (16.41, 26.72)	22.46 (16.43, 27.90)	− 0.56	0.58
ALB (g/L)	32.84 (28.83, 36.56)	33.69 (29.20, 37.00)	30.29 (27.55, 34.70)	− 2.62	**0.01**
Fer (ng/ml)	211.6 (90.1, 375.0)	211.6 (88.7, 375.0)	222.5 (102.2, 385.9)	− 0.02	0.99
HB(g/L)	82.62 ± 18.49	82.42 ± 18.13	83.55 ± 20.24	− 0.36	0.72
RBC (× 10^9^/L)	2.76 (2.32, 3.17)	2.67 (2.32, 317)	2.85 (2.34, 3.21)	− 0.62	0.53
GLU (mmol/L)	4.89 (4.16, 6.63)	4.89 (4.16, 6.28)	4.90 (4.14, 7.44)	− 0.42	0.68
Antithrombin III activity	97.9 (90.9, 105.7)	98.4 (92.9, 106.0)	93.90 (87.30, 106.50)	− 1.58	0.11
PTA	98.43 ± 20.29	99.95 ± 19.60	91.84 ± 22.14	2.09	**0.04**
PT (sec)	11.10 (10.60, 11.83)	11.00 (10.60, 11.72)	11.30 (10.50, 12.10)	− 0.62	0.53
PCT (ng/ml)	0.14 (0.06, 0.40)	0.14 (0.06, 0.40)	0.13 (0.05, 0.46)	− 0.33	0.74
Ca (mmol/L)	1.98 ± 0.33	2.00 ± 0.32	1.90 ± 0.38	1.77	0.08
P (mmol/L)	1.88 (1.46, 2.40)	1.84 (1.45, 2.37)	2.03 (1.63, 2.58)	− 1.64	0.10
Ca × Pi	44.77 (36.12, 56.25)	44.27 (35.79, 55.94)	50.59 (38.00, 58.51)	− 1.22	0.22
Creatinine (umol/L)	750.9 (604.5, 944.5)	750.9 (596.7, 946.8)	749.0 (609.8, 936.6)	− 0.12	0.91
Uric acid (umol/L)	487.32 ± 139.54	498.03 ± 136.78	439.41 ± 143.34	2.49	**0.01**
Urea nitrogen (mmol/L)	19.3 (23.6, 30.2)	23.5 (19.3, 30.8)	24.00 (19.09, 29.32)	− 0.04	0.97
K (mmol/L)	4.41 (3.90, 5.17)	4.37 (3.88, 5.12)	4.55 (3.91, 5.22)	− 0.90	0.37
eGFR (ml/min/1.73m^2^)	5.49 (3.99, 7.11)	5.41 (3.99, 7.11)	5.78 (3.89, 7.21)	− 0.39	0.70
D-dimer (ug/ml)	1.22 (0.62, 3.16)	1.09 (0.58, 2.82)	1.78 (0.87, 3.86)	− 1.68	0.09

*TC* total cholesterol, *TG* triglyceride, *HDL* high-density lipoprotein, *LDL* low-density lipoprotein, *PLT* platelets, *PDW* platelet distribution width, *MPV* mean platelet volume, *WBC* white blood cells, *PTH* parathyroid hormone, *β2MG* β2 microglobulin, *ALB* albumin, *Fer* ferritin, *HB* hemoglobin, *RBC* red blood cells, *GLU* glucose, *PTA* prothrombin activity, *PT* prothrombin time, *PCT* procalcitonin, *Ca* calcium, *P* phosphorus, *eGFR* estimated glomerular filtration rate.

Categorical variables are presented as number (%). Continuous variables with Gaussian distribution are presented as mean ± standard deviations;

P for comparison between patients with *AVF* patency and *AVF* dysfunction.

Significant values are in bold.

**Figure 3:**
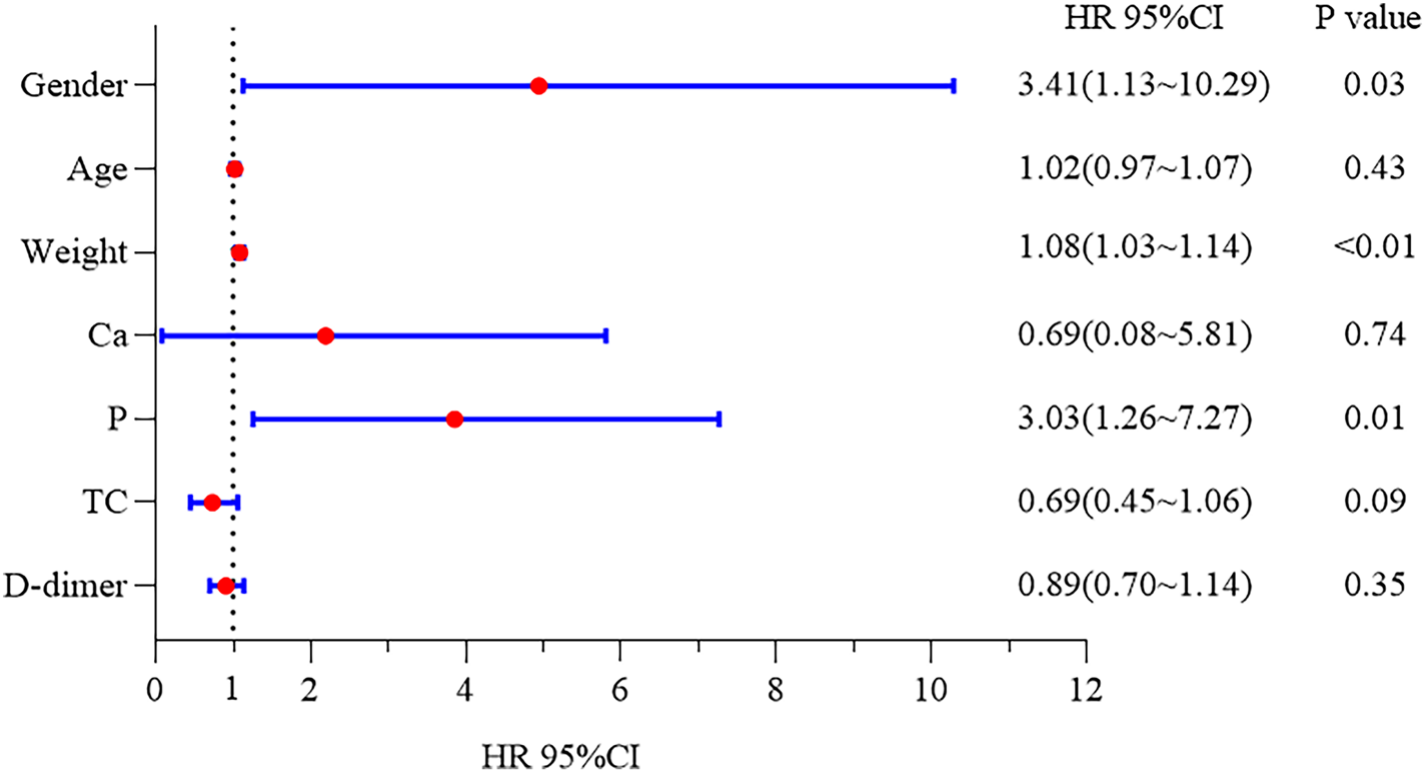
Forrest plot of risk factors of AVF dysfunction.

Univariate Cox regression analysis revealed that weight (HR, 1.03; *P* = 0.03) is a predictor for the loss of AVF function(Supplementary Table 1). In addition, multivariate Cox regression analysis further demonstrated that sex (HR, 3.41; *P* = 0.03) and weight (HR 1.08; *P* < 0.01) are independent risk factors for AVF failure (Table 3, Fig. 3). Although the difference in blood phosphorus level between the two groups was not statistically significant, multivariate Cox proportional hazard regression showed that blood phosphorus level is an independent risk factor for AVF failure (HR: 3.03; *P* = 0.01).

**Table 3:** Univariate and multivariate Cox proportional hazard regression analysis of risk factors associated with AVF dysfunction.

Characteristics	Univariate analysis					Multivariate analysis				
	B	P	HR	95% CI		B	P	HR	95% CI	
				Lower	Upper				Lower	Upper
Sex (female)	0.31	0.32	1.36	0.74	2.51	1.23	**0.03**	3.41	1.13	10.29
Age (year)	0.01	0.43	1.01	0.99	1.04	0.02	0.43	1.02	0.97	1.07
Weight (kg)	0.03	**0.03**	1.03	1.00	1.06	0.08	** < 0.01**	1.08	1.03	1.14
Ca (mmol/L)	− 0.80	0.06	0.45	0.19	1.05	− 0.37	0.74	0.69	0.08	5.81
P (mmol/L)	0.30	0.12	1.35	0.93	1.95	1.11	**0.01**	3.03	1.26	7.27
TC (mg/dl)	0.04	0.77	1.04	0.81	1.33	− 0.37	0.09	0.69	0.45	1.06
d-dimer (ug/ml)	0.03	0.55	1.03	0.94	1.12	− 0.11	0.35	0.89	0.70	1.14

Significant values are in bold.

*TC* total cholesterol, *Ca* calcium, *P* phosphorus.

## Discussion

The increasing demand for HD has been accompanied by a growing consensus on the “fistula first” approach among clinicians. The National Kidney Foundation Guidelines for Vascular Access, Fistula First Breakthrough Initiative (FFBI) established by Centers for Medicare and Medicaid (CMS), and other major renal societies across the globe recommend AVF as the preferred choice for patients requiring maintenance HD (MHD)^6–9^. Despite these recommendations, the utilization rate of AVF for dialysis initiation was reported to vary between 15 and 83%^10^. Furthermore, up to 20–60% of primary AVF failure can be observed in subsequent follow-ups^11^. A number of risk factors have been reported to affect the success of AVF. However, the detailed role of risk factors in AVF dysfunction remain elusive. This study revealed that sex, weight, and phosphorus level are independent risk factors associated with AVF dysfunction in Chinese ESRD patients, which is in line with previous report.

Hyperphosphatemia is the most common complication in MHD patients, and phosphorus is considered the initiator for vascular calcification. High level of extracellular inorganic phosphate (Pi) stimulates synthetic transformation of vascular smooth muscle cells (VSMCs) and induces the secretion of matrix vesicles to trigger apoptosis pathways, which contributes to VSMC failure and consequently progressive vascular calcification^12^. Vascular calcification accelerates vessel injury and thrombosis, ultimately leading to AVF failure. Several studies have reported a strong association between hyperphosphatemia and AVF failure. Zhou et al. showed that patients with hyperphosphatemia were more likely to develop AVF failure than their normal counterparts^13^. Similarly, Yu et al. found that blood Pi level was an independent risk factor for AVF failure^12^, which is consistent with our finding. Although our univariate analysis did not identify a significant correlation between blood phosphorus level and AVF failure, multivariate analysis revealed that hyperphosphatemia may be associated with a higher risk of AVF failure. Therefore, reducing positive phosphate balance and serum phosphate level by phosphate binders or regular HD may improve AVF patency in ESDR patients.

The impact of weight on AVA patency is currently unclear. Obesity-associated inflammation and advanced atherosclerosis may lead to endothelial injury, resulting in lower initial intraoperative blood flow and higher failure rate of primary AVF maturation^14^. The DOPPS reported that successful AVF placement was associated with a lower BMI^8^. Chan et al. reported that a BMI ≥ 35 kg/m^2^ significantly increased the risk of AVF failure by 3.66-fold^15^. Kim et al. revealed that obese patients had significantly longer maturation time and higher early maturation failure rate. A BMI ≥ 25 kg/m^2^ conferred a relative risk of 2.4 for AVF failure^16^. In line with these underlying mechanisms and previous clinical studies, we have discovered that increased weight may be correlated with a higher risk of AVF failure.

It has been convinced that gender profoundly contributes to AVF outcome. Our study suggested that female patients have a decreased AVF patency rate than men, which was in line with Bashar et al.’s finding that AVF non-maturation was associated with female gender. Several studies revealed that women have lower AVF maturation rates and longer maturation time than men^17,18^. Due to smaller mean vessel diameters in women, decreased vasodilation capacity, and insufficient outward remodeling^15,17^, female patients need more effort to salvage nonfunctioning AVFs to promote AVF maturation outcomes^19^.

Although diabetes is a well-established risk factor for advanced atherosclerosis and cardiovascular diseases, it was not a significant risk predictor for AVF failure in our study. A meta-analysis by Almasri et al. revealed that patients with diabetes and cardiovascular disease had a shorter duration of AVF patency compared with non-diabetic patients^17^. However, several studies were consistent with our findings, showing that there was no difference in AVF patency between diabetic and non-diabetic patients^20,21^.

In addition to the above parameters we included, a number of variables such as surgeon experience, AVF sites and vessel histology were associated with AVF maturation. The experience of surgeon is a vital cause for AVF success and patency. Fassiaadis et al. revealed that the primary AVF success was 13% higher when the senor surgeon who performed 15–18 AVF procedure per week, suggesting the impact of surgical experience on AVF maturation^22^. In addition, the sites of AVF created also affect the vascular access success. Sultan et al. has demonstrated distal placement of AVF need more intervention to salvage AVF patency and are linked to lower cumulative survival rate compared to those created proximally^23^. Venous diameter is considered to be an independent predictor for AVF maturation. The AVF maturation can be highly expected if the diameter of vein measured more than 4 mm in preoperative assessment, whereas AVF performed with vein with less than 2.5 mm in diameter had a high rate of non-maturation^24^. Moreover, Mendes et al. reported that 22 fistulae created with a cephalic vein less than 2 mm in diameter had 19 non-maturation, whereas 19 of 25 (76%) fistulae created using a cephalic vein diameter more than 2 mm successfully matured, verifying the predictive value of vein diameter in AVF failure^25^.

This study has several limitations. Firstly, this is a single-center retrospective study with a small sample size, which may have limited the statistical power of the analysis. Secondly, other factors that may influence AVF patency such as the status of blood vessels, surgical technique, AVF sites and vessel histology have not been included in our study. Therefore, further studies focusing on these parameters are warranted.

## Conclusion

AVF dysfunction is strongly associated with a number of risk factors including weight, phosphorus level, and sex. Thus, intervention strategies targeting these potential factors such as weight loss, or oral phosphate binders may improve the long-term success of AVF. Further multi-center randomized-controlled trials are needed to confirm these findings.

### Supplementary Information

Supplementary Table 1.

## Data Availability

The datasets used and analysed during the current study available from the corresponding author on reasonable request.
