# Supplementary material for: Risk factors for arteriovenous fistula dysfunction in hemodialysis patients: a retrospective study
Source: Sci Rep. 2023 Dec 3;13:21325. doi: 10.1038/s41598-023-48691-4 (PMC10694134; doi:10.1038/s41598-023-48691-4)
Supplement: Supplementary file 1 — Supplementary Table 1. [file 41598_2023_48691_MOESM1_ESM.docx]

**Supplementary Table1. Univariate Cox proportional hazard regression analysis**

**of risk factors associated with AVF dysfunction**

| Characteristics | Univariate analysis | | | | |  |
| --- | --- | --- | --- | --- | --- | --- |
|  | B | P | HR | 95%CI | |  |
|  |  |  |  | Lower | Upper |  |
| Sex | 0.31 | 0.32 | 1.36 | 0.74 | 2.51 |  |
| Age (year) | 0.01 | 0.43 | 1.01 | 0.99 | 1.04 |  |
| Weight（kg） | 0.03 | **0.03** | 1.03 | 1.00 | 1.06 |  |
| BMI | 0.03 | 0.67 | 1.03 | 0.90 | 1.18 |  |
| SBP (mmHg) | -0.04 | **<0.01** | 0.97 | 0.94 | 0.99 |  |
| DBP (mmHg) | -0.01 | 0.34 | 0.99 | 0.98 | 1.01 |  |
| Smoking | -0.66 | 0.27 | 0.52 | 0.16 | 1.67 |  |
| Cause of CKD |  |  |  |  |  |  |
| Glomerulonephritis |  | 0.90 |  |  |  |  |
| Others | 0.06 | 0.85 | 1.06 | 0.57 | 1.98 |  |
| Unkown | -0.39 | 0.71 | 0.68 | 0.09 | 5.08 |  |
| TC (mmol/L) | 0.04 | 0.77 | 1.04 | 0.81 | 1.33 |  |
| TG (mmol/L) | -0.05 | 0.77 | 0.95 | 0.66 | 1.36 |  |
| HDL (mmol/L) | 0.02 | 0.96 | 1.02 | 0.39 | 2.71 |  |
| LDL (mmol/L) | -0.10 | 0.58 | 0.91 | 0.65 | 1.27 |  |
| PLT (×10^9^/L) | 0.01 | 0.42 | 1.00 | 1.00 | 1.01 |  |
| MPV (fl) | 0.13 | 0.20 | 1.14 | 0.93 | 1.39 |  |
| PDW (fl) | -0.01 | 0.67 | 1.00 | 0.98 | 1.01 |  |
| WBC (×10^9^/L) | -0.02 | 0.73 | 0.98 | 0.86 | 1.11 |  |
| PTH (pg/ml) | 0.01 | 0.64 | 1.00 | 1.00 | 1.01 |  |
| NEU (×10^9^/L) | -0.02 | 0.73 | 0.98 | 0.85 | 1.12 |  |
| MONO (×10^9^/L) | 0.70 | 0.31 | 2.01 | 0.53 | 7.58 |  |
| LYMPH (×10^9^/L) | -0.19 | 0.58 | 0.83 | 0.42 | 1.64 |  |
| NLR | 0.01 | 0.77 | 1.01 | 0.95 | 1.08 |  |
| PLR | 0.01 | 0.32 | 1.00 | 1.00 | 1.01 |  |
| β2MG (mg/L) | 0.01 | 0.57 | 1.01 | 0.98 | 1.04 |  |
| ALB (g/L) | -0.07 | **0.01** | 0.93 | 0.88 | 0.99 |  |
| Fer (ng/ml) | 0.01 | 0.74 | 1.00 | 1.00 | 1.01 |  |
| HB(g/L) | 0.01 | 0.84 | 1.00 | 0.99 | 1.02 |  |
| RBC (×10^9^/L) | 0.09 | 0.69 | 1.10 | 0.70 | 1.71 |  |
| GLU (mmol/L) | 0.05 | 0.33 | 1.05 | 0.95 | 1.15 |  |
| Antithrombin III activity | -0.01 | 0.46 | 0.99 | 0.97 | 1.01 |  |
| PTA | -0.02 | **0.03** | 0.98 | 0.97 | 1.00 |  |
| PT (sec) | 0.02 | 0.92 | 1.02 | 0.76 | 1.36 |  |
| PCT (ng/ml) | 0.08 | 0.25 | 1.09 | 0.94 | 1.25 |  |
| Ca (mmol/L) | -0.80 | 0.06 | 0.45 | 0.19 | 1.05 |  |
| P（mmol/L） | 0.30 | 0.12 | 1.35 | 0.93 | 1.95 |  |
| Ca×Pi | 0.01 | 0.18 | 1.01 | 1.00 | 1.03 |  |
| Creatinine (umol/L) | 0.01 | 0.96 | 1.00 | 1.00 | 1.01 |  |
| Uric Acid (umol/L) | -0.01 | **0.02** | 1.00 | 1.00 | 1.00 |  |
| Urea nitrogen (mmol/L) | -0.01 | 0.95 | 1.00 | 0.97 | 1.03 |  |
| K (mmol/L) | 0.26 | 0.17 | 1.30 | 0.90 | 1.88 |  |
| EGFR | 0.02 | 0.67 | 1.02 | 0.93 | 1.13 |  |
| D-dimer (ug/ml) | 0.03 | 0.55 | 1.03 | 0.94 | 1.12 |  |

TC, total cholesterol; TG, triglyceride; HDL, high-density lipoprotein; LDL, low-density lipoprotein PLT, platelets; PDW, platelet distribution width; MPV, mean platelet volume; WBC, white blood cells; PTH, parathyroid hormone; β2MG, β2 microglobulin; ALB, albumin; Fer, ferritin; HB, hemoglobin; RBC, red blood cells; GLU, glucose; PTA,prothrombin activity;PT, prothrombin time; PCT, procalcitonin; Ca, calcium; P, phosphorus; eGFR, estimated glomerular filtration rate.
